# Radiomics Analysis of Non-Enhancing Lesions After Bevacizumab Administration in Recurrent Glioblastoma

**DOI:** 10.3390/bioengineering13010028

**Published:** 2025-12-26

**Authors:** Takahiro Sanada, Takeshi Shimizu, Yoshiko Okita, Hideyuki Arita, Hirotaka Sato, Masato Saito, Nobuyuki Mitsui, Satoru Hiroshima, Kayako Isohashi, Mishie Tanino, Yonehiro Kanemura, Haruhiko Kishima, Manabu Kinoshita

**Affiliations:** 1 Department of Neurosurgery, Asahikawa Medical University, Asahikawa 078-8510, Japan; kyokui100084@gmail.com (T.S.);; 2 Department of Neurosurgery, Japanese Red Cross Kitami Hospital, Kitami 090-8666, Japan; 3 Department of Neurosurgery, Osaka International Cancer Institute, Osaka 541-8567, Japan; 4 Department of Neurosurgery, Osaka University Graduate School of Medicine, Suita 565-0871, Japan; 5 Department of Neurosurgery, Faculty of Medicine, Miyazaki University, Miyazaki 889-1692, Japan; 6 Department of Diagnostic and Interventional Radiology, Osaka University Graduate School of Medicine, Suita 565-0871, Japan; 7 Department of Diagnostic Pathology, Asahikawa Medical University Hospital, Asahikawa 078-8510, Japan; 8 Department of Neurosurgery, NHO Osaka National Hospital, Osaka 540-0006, Japan; 9 Department of Biomedical Research and Innovation, Institute for Clinical Research, NHO Osaka National Hospital, Osaka 540-0006, Japan

**Keywords:** Glioblastoma, Bevacizumab, magnetic resonance image, radiomics, non-enhancing lesions, positron emission tomography

## Abstract

This study explored radiomic features that help identify non-contrast-enhancing tumors (nCET) by analyzing regions where contrast-enhancing tumors (CET) transformed into nCET after Bevacizumab (BEV) treatment. The BEV cohort included 24 recurrent GBM (rGBM) patients treated with BEV, showing reduced contrast-enhancement on gadolinium-enhanced T1-weighted imaging (T1Gd) imaging. The 11C-methionine positron emission tomography (Met-PET) cohort consisted of 24 newly diagnosed GBM (nGBM) patients with available Met-PET data. VOIs were created from T2WI, FLAIR, T1Gd, and Met-PET to analyze nCET and T2/FLAIR lesions. After significant radiomic features were identified, a prediction model for nCET was developed in the BEV cohort and subsequently evaluated in the Met-PET cohort. A total of 37 and 46 significant radiomic features were found in the BEV and Met-PET cohorts, respectively. The key feature, T2WI_whole_GLCMcorrelation_1, was selected for predictive modeling. The model demonstrated high accuracy (AUC = 0.93, *p* < 0.0001) in the BEV cohort, with sensitivity and specificity of 0.91, while the Met-PET cohort showed moderate accuracy (AUC = 0.74, *p* = 0.0053). Image reconstruction using these features also effectively visualized nCET in nGBM. These findings suggest that radiomic features in CET regions transforming to nCET after BEV treatment harbors valuable information for identifying nCET in GBM.

## 1. Introduction

The extent of tumor resection is one of the most significant prognostic factors for Glioblastoma (GBM) treatment [1,2,3]. Conventional maximum tumor resection focused on contrast-enhancing tumors (CET) as target lesions, but parts of the high-intensity regions on T2-weighted image or fluid-attenuated inversion recovery (T2/FLAIR) contain as many tumor cells as the CET [4], which is referred to as the non-contrast-enhancing tumor (nCET) [5]. Furthermore, not only CET’s but also nCET’s postoperative residual volume is reported to significantly impact GBM patients’ overall survival [6,7]. These findings highlight the necessity of developing a magnetic resonance imaging (MRI)-based method to differentiate between nCET and vasogenic edema in the high-intensity region on T2/FLAIR to improve the precision of presurgical planning [8,9]. However, amino acid positron emission tomography, such as 11C-methionine positron emission tomography (Met-PET), still remains the only reliable imaging modality to achieve this goal. Providing Met-PET as a gold standard imaging modality worldwide remains challenging due to the short half-life time of 11C and limited economic and physical accessibility. Hence, a novel MRI-based imaging method to visualize nCET is desired.

Bevacizumab (BEV) has been routinely administered for the treatment of recurrent GBM since its approval by the US Food and Drug Administration in 2009 [10]. Due to BEV’s antiangiogenic and blood–brain barrier stabilization effect, a radiographical phenomenon named “pseudoresponse” is occasionally observed, where Bevacizumab (BEV) transforms contrast-enhancing lesions into non-enhancing lesions with existing viable tumor cells without actual antitumor effect [11,12,13]. Therefore, lesions with diminished contrast enhancement after BEV administration could be considered equivalent to glioblastoma’s nCET. Under this assumption, the current study aimed to explore the possible use of radiomic features for identifying nCET by performing a radiomic analysis on areas where CETs transformed into nCET after BEV administration. Additionally, another cohort with Met-PET data was employed to determine the imaging features most predictive of nCET detection.

## 2. Materials and Methods

### 2.1. Patients and Cohorts

This study was performed in accordance with the principles of the Helsinki Declaration. It was approved by the internal ethical review boards of Asahikawa Medical University Hospital (AMUH, Approval number 21040) and collaborative institutions, including the Osaka International Cancer Institute (OICI) and Osaka University Hospital (OUH). Due to the retrospective nature of the study, the internal ethical review boards of AMUH waived the need to obtain informed consent. Two cohorts were prepared to explore radiomic features for identifying nCET: the BEV cohort from Asahikawa Medical University Hospital and the Met-PET cohort, which comprised data from Osaka International Cancer Institute and Osaka University Hospital. The patients in the Met-PET cohort did not receive BEV treatment at the time of data collection but underwent Met-PET, which served as the ground truth for detecting nCET.

The inclusion criteria for the present study were as follows: for the BEV cohort, we included recurrent GBM (rGBM) patients treated with intravenous BEV at a dose of 10 mg/kg or 15 mg/kg with a marked reduction in contrast-enhancement on T1WI-Gd following BEV administration. One case was excluded due to the unavailability of MR images before BEV administration. As a result, 24 rGBM patients were eligible. We included newly diagnosed GBM (nGBM) patients whose T1WI-Gd and Met-PET were available for the Met-PET cohort. One case was excluded due to the absence of Met-PET uptake and failure in imaging texture calculation. As a result, 24 nGBM patients were eligible for the Met-PET cohort.

### 2.2. Genetic Analysis and Pathological Diagnosis

Genetic analyses were performed at Osaka National Hospital according to the previously described procedures [14]. The presence of hotspot mutations in Isocitrate dehydrogenase (IDH1 R132 and IDH2 R172) was analyzed by Sanger sequencing. The pathological diagnosis was based on the 2016 World Health Organization Classification of Tumors of the Central Nervous System. Nine cases of the BEV cohort were diagnosed as “Glioblastoma, IDH-wildtype.” Fifteen cases with no information on the status of IDH mutation by the genetic analysis were classified as “Glioblastoma, NOS.” Among the 15 cases, 12 cases were confirmed as negative for IDH R132H mutation by immunochemistry, while immunochemistry was not performed in the remaining three cases. All cases in the Met-PET cohort were diagnosed as “Glioblastoma, IDH-wild type” based on genetic analysis.

**Figure 1:**
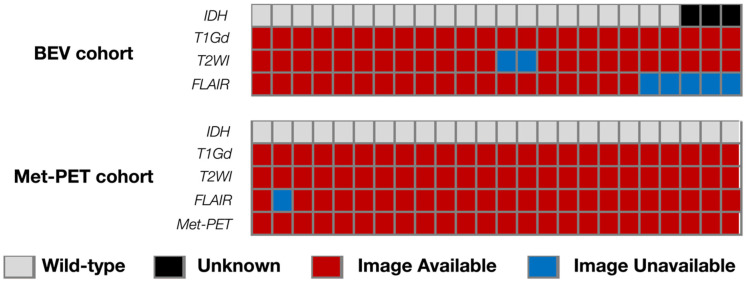
Overview of the analyzed cohort with the landscape of genetic information and availabilities of MRI sequences. The study prepared two cohorts: the BEV cohort with nCET after BEV administration and the Met-PET cohort with Met-PET.

Detailed information regarding the cohorts were provided in Figure 1, Supplementary Tables S1 and S2.

### 2.3. MRI and 11C-Methionine Positron Emission Tomography (Met-PET) Acquisition

All MRIs were acquired using either 1.5- or 3.0 T MRI scanners according to the protocols at AMUH for rGBM and at OICI and OUH for nGBM. Gd-T1WIs were available for all cases. On the other hand, fluid-attenuated inversion recovery (FLAIR) images of both before and after BEV administration were available in 19 cases, and T2-weighted images (T2WI) were available in 22 cases for the BEV cohort. FLAIR images were available in 23 cases and T2WI in all cases for the Met-PET cohort.

PET studies were performed using an Eminence-G system (Shimadzu, Kyoto, Japan), with 11C-Methionine synthesized according to the previous method [15] and injected intravenously at a dose of 3 MBq/kg body weight. Tracer accumulation was recorded in trans-axial sections over the entire brain, and the summed activity (the standard uptake value: SUV) from 20 to 32 min after tracer injection was used for image reconstruction. Images were stored in 256 by 256 by 59 or 99 anisotropic voxels, with each voxel being 1 by 1 by 2.6 mm.

All image data in Digital Imaging and Communication in Medicine (DICOM) format were converted into Neuroimaging Informatics Technology Initiative (NIfTI) format using Mango software (version 4.0.1; University of Texas Health Science Center, https://mangoviewer.com/mango.html, accessed on 22 August 2024).

### 2.4. The Concept of Crafting Voxels-of-Interest

**Figure 2:**
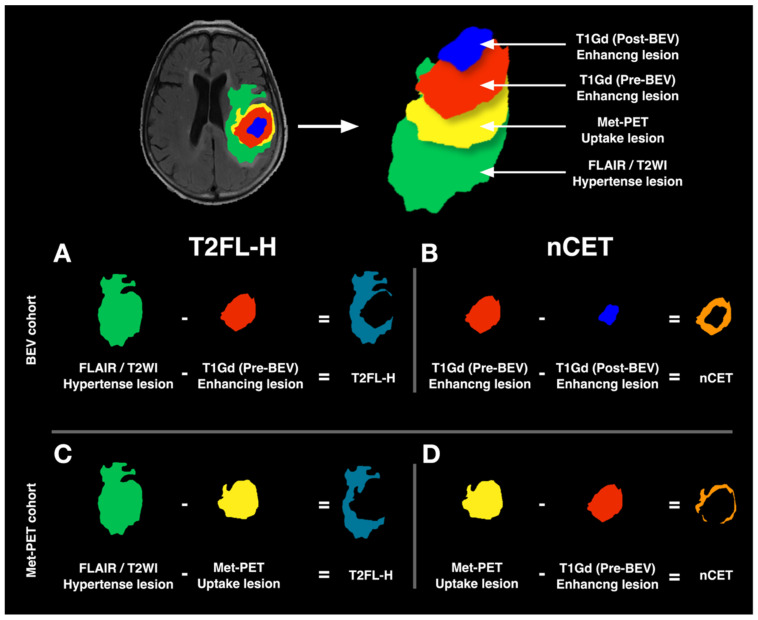
Schema of VOI creation. Four types of VOIs were generated based on the Gd enhancement of glioblastomas before and after BEV administration (Pre-BEV and Post-BEV), T2WI/FLAIR hyperintense lesions, and Met-PET uptake lesions. In the BEV cohort, VOIs on pre-BEV T1Gd were subtracted from Pre-BEV T2WI or FLAIR to obtain the non-contrast-enhancing T2/FLAIR lesion (T2FL-H or VOI_T2FL-H_) (**A**). VOIs on Gd-T1WI Post-BEV were then subtracted from the VOIs on T1Gd Pre-BEV to determine the lesion in which CET converted to nCET after BEV (nCET or VOI_nCET_) (**B**). In the Met-PET cohort, subtractions of Met-PET from T2WI/FLAIR VOIs yielded the VOI_T2FL-H_ (**C**), and subtractions of T1Gd from Met-PET VOIs yielded the VOI_nCET_ (**D**).

Volumes-of-interests (VOI)s were created from T2WI, FLAIR, and T1Gd acquired pre- and post-BEV administration for the BEV cohort. VOIs on pre-BEV T1Gd were subtracted from pre-BEV T2WI or FLAIR to obtain the non-contrast-enhancing T2/FLAIR lesion (T2FL-H or VOI_T2FL-H_, Figure 2A). VOIs on Gd-T1WI post-BEV were then subtracted from the VOIs on T1Gd pre-BEV to determine the lesion in which CET converted to nCET after BEV (VOI_nCET_, Figure 2B). On the other hand, T2WI, FLAIR, T1Gd, and Met-PET were used to create VOIs for the Met-PET cohort. VOIs of Met-PET were segmented under visual identification of abnormal tracer uptake. Subtractions of Met-PET from T2WI/FLAIR VOIs yielded the VOI_T2FL-H_ (Figure 2C), and subtractions of T1Gd from Met-PET VOIs yielded the VOI_nCET_ (Figure 2D).

### 2.5. Image Co-Registration and Radiomics

Every sequence obtained and the VOIs mentioned above from a single subject was co-registered to FLAIR or T2WI, based on the NIfTI “general affine transformation” coordinate system utilizing the Volume Imaging in Neurological Research, Co-Registration and (VINCI; Max Planck Institute for Neurological Research Cologne, Germany, https://vinci.sf.mpg.de, accessed on 22 August 2024). Author 1, with 9 years of experience board certified by the Japan Neurosurgical Society, manually handcrafted VOIs by using the ITK-SNAP software (version 3.8.0, http://www.itksnap.org/pmwiki/pmwiki.php, accessed on 22 August 2024).

Image features were extracted from FLAIR and T2WI according to the previously described method [14,16]. FLAIR and T2WI were converted into 256-level grayscale images after cutting off the upper 0.1% signal. This procedure was necessary for intensity normalization across all images acquired by different MRI scanners [16]. VOI_T2FL-H_ was applied to FLAIR and T2WI before BEV administration, while VOI_nCET_ was applied to those in the BEV cohort after BEV administration. VOI_T2FL-H_ and VOI_nCET_ were applied to FLAIR and T2WI without receiving any treatment for the Met-PET cohort. First-order texture features were calculated based on histograms of the 256-level grayscale within each VOI applied on FLAIR and T2WI. Second-order texture features were measured using the Gray Level Co-occurrence Matrix (GLCM) and Gray Lebel Run Length Matrix (GLRLM). In total, 49 radiomic features were extracted from each image. Details of the extracted radiomic features have been provided previously [16]. Workflow across the entire study is described in Supplementary Figure S1.

### 2.6. Statistical Analysis and Imaging Feature Selection

Statistical analysis was performed using Prism 9 for Mac OS (GraphPad Software, San Diego, CA, USA). The difference in each radiomic feature between VOI_T2FL-H_ and VOI_nCET_ was investigated by the Mann–Whitney U test and receiver-operating characteristic (ROC) curve analysis. A *p*-value of less than 0.05 was considered significant. Benjamini–Hochberg (BH) test confirmed the FDR (False Discovery Rate) with BH-adjusted *p* value of less than 0.05. Subsequently, significant radiomic features were identified within each cohort and subsequently ranked according to the mean area under the ROC curve (AUC) across both cohorts. The mean AUC served as a metric to assess the overall diagnostic performance of the radiomic features. Radiomic features were further selected based on the similarity of the median value trends of VOI_T2FL-H_ and VOI_nCET_ between both cohorts. For example, if a radiomic feature’s median value of VOI_T2FL-H_ is greater than that of VOI_nCET_ in one cohort, a similar trend should be observed in the other. Finally, the three highest-raking features were chosen for subsequent multiple regression analysis, considering the requirement of at least ten events per variable in multiple logistic regression analysis [17]. Finally, variance inflation analysis (VIF) confirmed the decorrelation of the variances, selecting the final variances for the model. Car package version 0.15 for R with default parameters was used for this analysis.

### 2.7. The Evaluation of nCET Prediction Through Radiomic Features in Newly Diagnosed GBM

ROC curve analysis using the identified the radiomic feature was performed in both the BEV and Met-PET cohorts. The cut-off value of the radiomic feature in BEV cohort was subsequently applied to the Met-PET cohort, where values were classified as either VOI_T2FL-H_ or VOI_nCET_. Sensitivity, specificity, positive predictive value (PPV), and negative predictive value (NPV) were calculated to evaluate the model’s diagnostic performance.

### 2.8. nCET Predictive Image Reconstruction via Radiomics

The identified radiomic feature was used to reconstruct an image highlighting nCET risk areas. T2WI were normalized and converted into 256-level grayscale images after cutting off the upper 0.1% signal. The images were segmented using kernel sizes of 8, 12 and 16 to ensure a clear division of pixels, and the identified radiomic feature from the above analysis were calculated within each kernel. The predicted values for each kernel were calculated using the identified radiomic feature. The reconstructed images highlighting nCET were applied to the VOI of segmented FLAIR and overlaid on the original FLAIR images. The final reconstructed image was visually compared with Met-PET in representative cases from the Met-PET cohort.

## 3. Results

### 3.1. Significant Radiomic Features for VOI_nCET_

**Figure 3:**
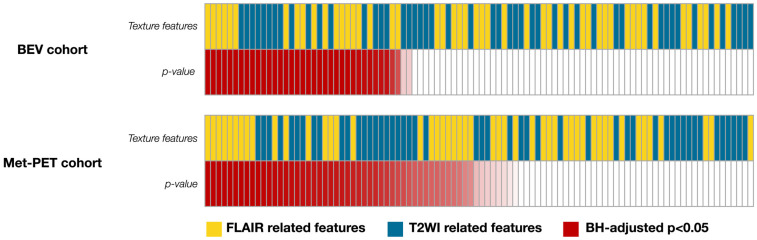
An overview of radiomic features and *p*-values in identifying nCET are described. Thirty-seven radiomic features significantly differed between VOI_nCET_ and VOI_T2FL-H_ within the BEV cohort, while forty-six features significantly differed within the Met-PET cohort. Statistically significant *p*-values are highlighted in red with a gradation, where smaller BH-adjusted *p*-values are represented by a darker shade of red (BH-adjusted *p* < 0.05).

Thirty-seven radiomic features significantly differed between VOI_nCET_ and VOI_T2FL-H_ within the BEV cohort, comprising 17 features from FLAIR and 18 from T2WI (Figure 3). On the other hand, 46 features differed significantly, including 23 from FLAIR and 23 from T2WI within the Met-PET cohort (Figure 3). Notably, 10 radiomic features were significant in both cohorts. Among these, T2WI_whole_GLCMcorrelation_1, FLAIR_whole_GLCMcorrelation_1 and FLAIR_whole_GLCMcorrelation_2 demonstrated higher rankings based on the mean AUC across both cohorts. These features consistently showed a similar relationship in their median values between T2FL-H and nCET between cohorts (Table 1), leading subsequent multiple logistic regression analysis. For example, the median values of “FLAIR_whole_GLCMcorrelation_1” of T2FL-H were higher than that of nCET in both cohorts. The mean AUC, individual AUC, *p*-values, and median values of the other significant radiomic features are detailed in Supplementary Table S3. Finally, VIF analysis showed the correlation of the features; T2WI_whole_GLCMcorrelation_1: 13.2, FLAIR_whole_GLCMcorrelation_1: 122.9, FLAIR_whole_GLCMcorrelation_2, 131.7. Therefore, T2WI_whole_GLCMcorrelation_1 was only selected for the subsequent analysis.

### 3.2. The Predictive Model for nCET in the BEV Cohort

**Figure 4:**
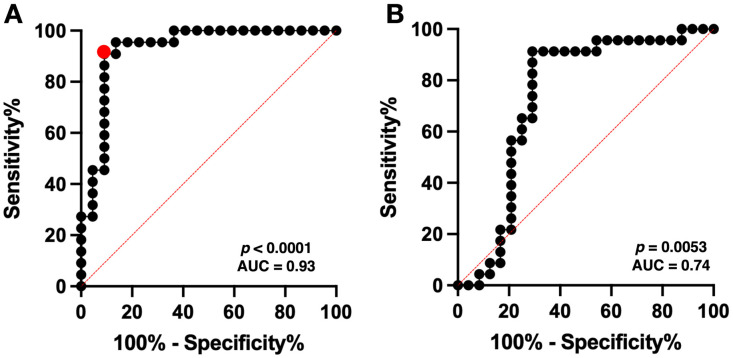
The predictive equation from three significant radiomic features demonstrated a high accuracy with an area under the ROC curve (AUC) of 0.93 (*p* < 0.0001) in the BEV cohort, with optimal cut-off values providing a sensitivity of 0.91 and specificity of 0.91 (red circle) (**A**). ROC curve analysis of the Met-PET cohort showed an AUC of 0.74 (*p* = 0.0053) with a sensitivity of 0.57–0.65 and a specificity of 0.71–0.79, using the obtained cut-off value for detecting nCET from the Met-PET cohort (**B**). The red dotted line indicates the line of AUC = 50%.

ROC curve analysis using T2WI_whole_GLCMcorrelation_1 revealed the optimal cut-off value to be 0.8917–0.9072, with a sensitivity of 0.91 and a specificity of 0.91 (AUC = 0.93, *p* < 0.0001) (Figure 4A).

### 3.3. The Predictive Model for nCET in Newly Diagnosed GBM in the Met-PET Cohort

ROC curve analysis of the Met-PET cohort showed an AUC of 0.74 (*p* = 0.0053, Figure 4B) with a sensitivity of 0.57–0.65 and a specificity of 0.71–0.79, using the previously obtained cut-off value for detecting nCET in the BEV cohort. Subsequently, the predictive performance using the cut-off value of in BEV cohort was evaluated for a total of 47 VOIs from the Met-PET cohort, excluding two VOIs due to failure in calculating the GLCM correlation of nCET. Fisher’s exact test showed significant difference in the Met-PET cohort (*p* = 0.0087, odds ratio = 5.55, 95% CI = 1.52–17.01). The sensitivity and specificity of the model in predicting nCET in nGBMs were 0.70 and 0.71. Additionally, positive predictive value (PPV) and negative predictive value (NPV) were 0.70 and 0.71.

### 3.4. Reconstruction of nCET Predictive Image from Radiomic Features

**Figure 5:**
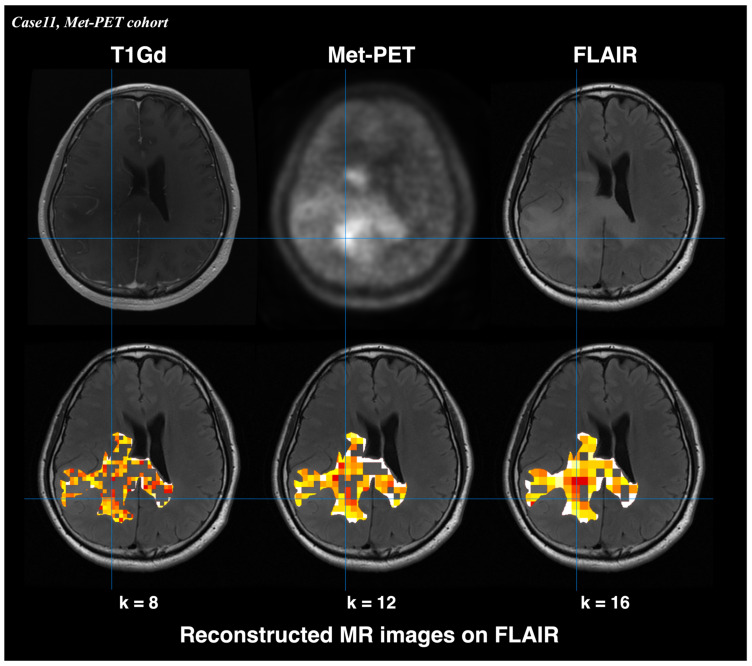
Reconstructed MR images derived from T2WI_whole_GLCMcorrelation_1 showed visualization of nCET in nGBM. High-risk regions corresponded to areas of increased uptake on Met-PET. Lower T2WI_whole_GLCMcorrelation_1 values indicate higher nCET risk. The color scale represents T2WI_whole_GLCMcorrelation_1 value (red: low value [high nCET risk]; white: high value [low nCET risk]). Met-PET high-uptake areas demonstrated nCET risk values between 0 and 0.78, the highest value.

Reconstructed MR images from T2WI_whole_GLCMcorrelation_1 enabled visualization of nCET in nGBM in the representative case (Figure 5). Regions with lower T2WI_whole_GLCMcorrelation_1 value—reflecting higher nCET risk—corresponded to areas of increased Met-PET uptake. Met-PET high-uptake areas demonstrated nCET risk values between 0 and 0.78, the highest value among those achieving a specificity of 1.00 (sensitivity: 0.27) in the ROC analysis of the BEV cohort.

## 4. Discussion

The CET has historically been the primary target for surgical resection that positively impacts the prognosis of nGBM [1,2]. On the other hand, it has long been known that tumor cells exist beyond CET in the high-intensity regions on T2/FLAIR, referred to as the nCET [4]. Thus, the surgical target is now shifting from CET to nCET, referred to as a “supratotal resection.” More importantly, complete resection of nCET is suggested to improve nGBM’s prognosis [3,6,7,18,19]. Previous stereotactic tissue sampling reach showed that Met-PET closely correlated with glioma cell density and exhibited superior diagnostic accuracy compared to conventional MRI [4,20,21]. Consequently, Met-PET is regarded as the gold standard for nCET identification. However, a novel MRI-based imaging method to visualize nCET as an alternative to Met-PET is necessary due to challenges in integrating Met-PET into routine clinical practice, hindered by limited accessibility and its economic constraints.

Interestingly, we often observe a phenomenon where contrast-enhancing lesions become non-enhancing following BEV treatment. This decrease in enhancement is frequently referred to as a “pseudoresponse,” which is attributed to BEV’s antiangiogenic effect and stabilization of the blood–brain barrier (BBB) without exerting an actual antitumor effect [11,12,13]. Therefore, these non-enhancing lesions, which transformed from enhanced lesions by BEV, could be considered equivalent to GBM’s nCET. Therefore, we explored radiomic features that enable the identification of nCET using conventional MRI by radiomic analysis of the area of decreased enhancement after BEV administration.

The present study identified the radiomic features—T2WI_whole_GLCMcorrelation_1—as the most promising predictors of nCET across both cohorts. ROC curve analysis using the radiomic feature showed achieved a high AUC of 0.93 (*p* < 0.0001) in the BEV cohort and a moderate high AUC of 0.74 (*p* = 0.0053), highlighting the radiomic feature’s potential utility for distinguishing nCET within T2/FLAIR high-intensity areas. The identified GLCM correlation textures are second-order features that reflect the spatial relationships between gray-level values of pixels or voxels [22]. Our results showed that the GLCM correlation texture of nCET was significantly lower than non-nCET T2/FLAIR high-intensity lesions, suggesting a more significant inhomogeneity in nCET than in non-nCET T2/FLAIR high-intensity lesions. This phenomenon could be due to differences in cellular composition and microenvironment of the affected tissue. These findings align with similar texture analysis studies, which demonstrated the GLCM texture values of T2WI and FLAIR being significantly lower in true progression compared to pseudoprogression in GBM [23]. While previous studies demonstrated the utility of qualitative MRI features for predicting nCET [8], this study is the first to quantitatively identify MRI features distinguishing nCET from non-nCET T2/FLAIR high-intensity lesions with high accuracy (AUC = 0.93) in the BEV cohort. We also succeeded in reversing radiomic features into observable and interpretable images to visualize nCET in nGBM. The developed model’s only modest PPV of 0.70 and NPV of 0.71 for the Met-PET cohort could be explained by the fact that the Met-PET cohort used a different ground truth from the BEV cohort. Nevertheless, the present study is the first to demonstrate radiomics features in CET regions transforming to nCET after BEV administration, harboring significant information useful for identifying nCET in GBM.

Several limitations of the present study must be addressed. First, the sample size of the cohort was small, with cases missing several MRI sequences. The inhomogeneous data could have affected the process during radiomic feature selection for detecting nCET. Ideally, a cohort with a larger sample size and a complete MRI sequence is desirable to validate our findings. Second, 15 cases in the BEV cohort lacked information on the IDH mutation status based on genomic analysis and thus were classified as “Glioblastoma, NOS.” While 12 cases were immunochemically confirmed as IDH R132H mutation negative, the remaining three were not. Although the lack of genetic tumor characterization could represent a limitation, it was unlikely to substantially affect the main conclusions of the present study, as our primary objective was to explore the radiomic features of nCET independent of molecular stratification. Third, the high accuracy observed in the BEV cohort may be attributed to overfitting, as the developed model was applied to one of the two native cohorts. Further external validation with an independent cohort is warranted to confirm the findings of the present study. Forth, due to the retrospective nature of this study, advanced MRIs, such as diffusion imaging and perfusion imaging, could not be integrated. A prospective intraoperative study incorporating state-of-the-art MRI sequences with histopathological samplings is desired to confirm the present study’s findings. Furthermore, although this study adopted manually delineated VOIs by a single investigator [24,25] to ensure consistent VOI definition across multiple imaging sequences in all cases, future studies incorporating multi-rater segmentation and formal reproducibility analyses would further strengthen the robustness of our findings.

## 5. Conclusions

In conclusion, the present study identified significant radiomic features for detecting nCET in GBM by performing radiomic analysis on areas where CETs transformed into nCET after BEV administration. The predictive model and reconstructed images from the radiomic features could help provide a reasonable visualization of nCET in GBM.

## Figures and Tables

**Table 1:** AUC and *p*-values (BH-adjusted *p*-values) of the imaging features in the BEV cohort from AMUH and in the Met-PET cohort from OSAKA.

Imaging Features	Mean AUC	BEV Cohort					Met-PET Cohort				
		ROC Analysis	BH-Adjusted *p* Value	Mann–Whitney U Test			ROC Analysis	BH-Adjusted *p* Value	Mann–Whitney U Test		
		AUC		*p* Value	Median		AUC		*p* Value	Median	
					T2FLH	nCET				T2FLH	nCET
**T2WI_whole_GLCMcorrelation_1**	0.833	0.928	*p* < 0.001 ^†^	*p* < 0.001 ^†^	0.942	0.843	0.737	0.019 ^†^	0.005 ^†^	0.931	0.886
**FLAIR_whole_GLCMcorrelation_1**	0.831	0.957	*p* < 0.001 ^†^	*p* < 0.001 ^†^	0.945	0.848	0.706	0.041 ^†^	0.018 ^†^	0.931	0.897
**FLAIR_whole_GLCMcorrelation_2**	0.826	0.951	*p* < 0.001 ^†^	*p* < 0.001 ^†^	0.883	0.714	0.702	0.044 ^†^	0.020 ^†^	0.860	0.798

^†^ indicates statistical significance with *p* < 0.05 when comparing radiomic featreus deriving from T2FLH and nCET.

## Data Availability

The original contributions presented in the study are included in the article/Supplementary Materials, further inquiries can be directed to the corresponding author/s.

## Supplementary Materials

The following supporting information can be downloaded at https://www.mdpi.com/article/10.3390/bioengineering13010028/s1, Table S1: Detailed demographic data regarding the BEV cohort; Table S2: Detailed demographic data regarding the Met-PET cohort; Table S3: The mean AUC, individual AUC, BH-adjusted *p*-values, *p*-values, and median values of the significant radiomic features across both cohorts; Figure S1: Workflow for image analysis and radiomics. Raw analyzed data in the BEV cohort and the Met-PET cohort are available in Supplementary Dataset S1. Correspondence and requests for materials should be addressed to M.K.

## Abbreviations

The following abbreviations are used in this manuscript:

nCET	Non-contrast-enhancing tumors
CET	Contrast-enhancing tumors
BEV	Bevacizumab
GBM	Glioblastoma
rGBM	Recurrent GBM
nGBM	Newly diagnosed GBM
MRI	Magnetic resonance imaging
T1Gd	Gadolinium-um-enhanced T1-weighted imaging
FLAIR	Fluid-attenuated inversion recovery
T2WI	T2-weighted images
IDH	Isocitrate dehydrogenase
Met-PET	11C-methionine positron emission tomography
AMUH	Asahikawa Medical University Hospital
OICI	Osaka International Cancer Institute
OUH	Osaka University Hospital
GLCM	Gray Level Co-occurrence Matrix
GLRLM	Gray Lebel Run Length Matrix
PPV	Positive predictive value
NPV	Negative predictive value
ROC	receiver-operating characteristic
AUC	Area under the curve

## References

[1] Sanai N., Polley M.-Y., McDermott M.W., Parsa A.T., Berger M.S. (2011). An Extent of Resection Threshold for Newly Diagnosed Glioblastomas. J. Neurosurg..

[2] Lacroix M., Abi-Said D., Fourney D.R., Gokaslan Z.L., Shi W., DeMonte F., Lang F.F., McCutcheon I.E., Hassenbusch S.J., Holland E. (2001). A Multivariate Analysis of 416 Patients with Glioblastoma Multiforme: Prognosis, Extent of Resection, and Survival. J. Neurosurg..

[3] Li Y.M., Suki D., Hess K., Sawaya R. (2016). The Influence of Maximum Safe Resection of Glioblastoma on Survival in 1229 Patients: Can We Do Better than Gross-Total Resection?. J. Neurosurg..

[4] Kinoshita M., Arita H., Okita Y., Kagawa N., Kishima H., Hashimoto N., Tanaka H., Watanabe Y., Shimosegawa E., Hatazawa J. (2016). Comparison of Diffusion Tensor Imaging and (11)C-Methionine Positron Emission Tomography for Reliable Prediction of Tumor Cell Density in Gliomas. J. Neurosurg..

[5] Lasocki A., Gaillard F. (2019). Non-Contrast-Enhancing Tumor: A New Frontier in Glioblastoma Research. Am. J. Neuroradiol..

[6] Karschnia P., Young J.S., Dono A., Häni L., Sciortino T., Bruno F., Juenger S.T., Teske N., Morshed R.A., Haddad A.F. (2022). Prognostic Validation of a New Classification System for Extent of Resection in Glioblastoma: A Report of the RANO Resect Group. Neuro-Oncology.

[7] Molinaro A.M., Hervey-Jumper S., Morshed R.A., Young J., Han S.J., Chunduru P., Zhang Y., Phillips J.J., Shai A., Lafontaine M. (2020). Association of Maximal Extent of Resection of Contrast-Enhanced and Non–Contrast-Enhanced Tumor with Survival Within Molecular Subgroups of Patients with Newly Diagnosed Glioblastoma. JAMA Oncol..

[8] Yamamoto S., Okita Y., Arita H., Sanada T., Sakai M., Arisawa A., Kagawa N., Shimosegawa E., Nakanishi K., Kinoshita M. (2023). Qualitative MR Features to Identify Non-Enhancing Tumors within Glioblastoma’s T2-FLAIR Hyperintense Lesions. J. Neurooncol..

[9] Akbari H., Kazerooni A.F., Ware J.B., Mamourian E., Anderson H., Guiry S., Sako C., Raymond C., Yao J., Brem S. (2021). Quantification of Tumor Microenvironment Acidity in Glioblastoma Using Principal Component Analysis of Dynamic Susceptibility Contrast Enhanced MR Imaging. Sci. Rep..

[10] Cohen M.H., Shen Y.L., Keegan P., Pazdur R. (2009). FDA Drug Approval Summary: Bevacizumab (Avastin^®^) as Treatment of Recurrent Glioblastoma Multiforme. Oncologist.

[11] Brandsma D., Bent M.J. (2009). van den Pseudoprogression and Pseudoresponse in the Treatment of Gliomas. Curr. Opin. Neurol..

[12] Nowosielski M., Wen P. (2018). Imaging Criteria in Neuro-Oncology. Semin. Neurol..

[13] Arevalo O.D., Soto C., Rabiei P., Kamali A., Ballester L.Y., Esquenazi Y., Zhu J.-J., Riascos R.F. (2019). Assessment of Glioblastoma Response in the Era of Bevacizumab: Longstanding and Emergent Challenges in the Imaging Evaluation of Pseudoresponse. Front. Neurol..

[14] Sanada T., Yamamoto S., Sakai M., Umehara T., Sato H., Saito M., Mitsui N., Hiroshima S., Anei R., Kanemura Y. (2022). Correlation of T1- to T2-Weighted Signal Intensity Ratio with T1- and T2-Relaxation Time and IDH Mutation Status in Glioma. Sci. Rep..

[15] Hatakeyama T., Kawai N., Nishiyama Y., Yamamoto Y., Sasakawa Y., Ichikawa T., Tamiya T. (2008). 11C-Methionine (MET) and 18F-Fluorothymidine (FLT) PET in Patients with Newly Diagnosed Glioma. Eur. J. Nucl. Med..

[16] Sasaki T., Kinoshita M., Fujita K., Fukai J., Hayashi N., Uematsu Y., Okita Y., Nonaka M., Moriuchi S., Uda T. (2019). Radiomics and MGMT Promoter Methylation for Prognostication of Newly Diagnosed Glioblastoma. Sci. Rep..

[17] Peduzzi P., Concato J., Kemper E., Holford T.R., Feinstein A.R. (1996). A Simulation Study of the Number of Events per Variable in Logistic Regression Analysis. J. Clin. Epidemiol..

[18] Incekara F., Smits M., van der Voort S.R., Dubbink H.J., Atmodimedjo P.N., Kros J.M., Vincent A.J.P.E., Bent M. (2020). van den The Association Between the Extent of Glioblastoma Resection and Survival in Light of MGMT Promoter Methylation in 326 Patients with Newly Diagnosed IDH-Wildtype Glioblastoma. Front. Oncol..

[19] de Leeuw C.N., Vogelbaum M.A. (2019). Supratotal Resection in Glioma: A Systematic Review. Neuro-Oncology.

[20] Kracht L.W., Miletic H., Busch S., Jacobs A.H., Voges J., Hoevels M., Klein J.C., Herholz K., Heiss W.-D. (2004). Delineation of Brain Tumor Extent with [11C]l-Methionine Positron Emission Tomography: Local Comparison with Stereotactic Histopathology. Clin. Cancer Res..

[21] Verburg N., Hoefnagels F.W.A., Barkhof F., Boellaard R., Goldman S., Guo J., Heimans J.J., Hoekstra O.S., Jain R., Kinoshita M. (2017). Diagnostic Accuracy of Neuroimaging to Delineate Diffuse Gliomas within the Brain: A Meta-Analysis. Am. J. Neuroradiol..

[22] Haralick R.M., Shanmugam K., Dinstein I. (1973). Textural Features for Image Classification. IEEE Trans. Syst. Man Cybern..

[23] Chen X., Wei X., Zhang Z., Yang R., Zhu Y., Jiang X. (2015). Differentiation of True-Progression from Pseudoprogression in Glioblastoma Treated with Radiation Therapy and Concomitant Temozolomide by GLCM Texture Analysis of Conventional MRI. Clin. Imaging.

[24] Pignotti F., Ius T., Russo R., Bagatto D., Bartoli F.B., Boccia E., Boldrini L., Chiesa S., Ciardi C., Cusumano D. (2024). Development and Validation of a MRI-Radiomics-Based Machine Learning Approach in High Grade Glioma to Detect Early Recurrence. Front. Oncol..

[25] Ingrisch M., Schneider M.J., Nörenberg D., de Figueiredo G.N., Maier-Hein K., Suchorska B., Schüller U., Albert N., Brückmann H., Reiser M. (2017). Radiomic Analysis Reveals Prognostic Information in T1-Weighted Baseline Magnetic Resonance Imaging in Patients with Glioblastoma. Investig. Radiol..

