# Supplementary material for: Radiomics Analysis of Non-Enhancing Lesions After Bevacizumab Administration in Recurrent Glioblastoma
Source: Bioengineering (Basel). 2025 Dec 26;13(1):28. doi: 10.3390/bioengineering13010028 (PMC12837343; doi:10.3390/bioengineering13010028)
Supplement: Supplementary file 1 [file bioengineering-13-00028-s001.zip › bioengineering-4005487-supplementary/bioengineering-4005487-supplementary/Supplementary Figure S1 Legend.pdf]

Supplementary Figure S1. Workflow for image analysis and radiomics.

Intensity normalization was performed across all images. In the BEV cohort, three types of VOIs were created based on FLAIR hyperintense lesions and Gd-enhancing lesions before and after BEV administration (Pre-BEV and Post-BEV). In the Met-PET cohort, three types of VOIs were created based on FLAIR hyperintense lesions and Gd-enhancing lesions. All VOIs were co-registered, and VOIs of T2FL-H and nCET were subsequently calculated. The VOIs of T2FL-H were applied to images obtained before BEV administration, while the VOIs for nCET were applied to images obtained after BEV administration. Finally, first-order and second-order texture analyses were performed.
