# Supplementary material for: Radiomics Analysis of Non-Enhancing Lesions After Bevacizumab Administration in Recurrent Glioblastoma
Source: Bioengineering (Basel). 2025 Dec 26;13(1):28. doi: 10.3390/bioengineering13010028 (PMC12837343; doi:10.3390/bioengineering13010028)
Supplement: Supplementary file 1 [file bioengineering-13-00028-s001.zip › bioengineering-4005487-supplementary/bioengineering-4005487-supplementary/Supplementary Table S1.pdf]

Supplementary Table S1. Detailed demographic data for the BEV cohort.

Image ID	Sex	Age	Lesion	Diagnosis	BEV inufusions, n	IDH (wt:1, unknown:0)	IDH mutation diagnosis	Reference	FLAIR (Yes:1, No:0)	T2IW (Yes:1, No:0)	Comment
00001	F	71	Frontal	GBM, IDH-wt	12	1	Sanger sequencing	FLAIR	1	1	
00002	M	72	Frontal	GBM, IDH-wt	2	1	Sanger sequencing	FLAIR	1	1	
00003	M	49	Temporal	GBM, IDH-wt	7	1	Sanger sequencing	FLAIR	1	1	
00004	M	47	Frontal, Parietal	GBM, IDH-wt	3	1	Sanger sequencing	FLAIR	1	1	
00005	M	55	Occipital	GBM, IDH-wt	3	1	Sanger sequencing	FLAIR	1	1	Excluded
00006	M	64	Temporal	GBM, IDH-wt	1	1	Sanger sequencing	FLAIR	1	1	
00007	F	80	Frontal, Cerebellum	GBM, IDH-wt	1	1	Sanger sequencing	FLAIR	1	0	
00008	F	62	Caudate	GBM, NOS	1	1	Immunostainig	FLAIR	1	1	
00009	F	68	Frontal	GBM, NOS	4	1	Immunostainig	FLAIR	1	1	
00010	M	64	Parietal	GBM, NOS	1	1	Immunostainig	FLAIR	1	1	
00011	F	64	Cerebellum, Brain stem	GBM, NOS	2	1	Immunostainig	FLAIR	1	1	
00012	F	67	Frontal, Parietal	GBM, NOS	1	1	Immunostainig	T2WI	0	1	
00013	F	65	Frontal, temporal	GBM, NOS	3	1	Immunostainig	FLAIR	1	0	
00014	M	70	Parietal	GBM, NOS	5	0		T2WI	0	1	
00015	F	73	Temporal	GBM, NOS	4	1	Immunostainig	FLAIR	1	1	
00016	F	72	Temporal	GBM, NOS	3	1	Immunostainig	FLAIR	1	1	
00017	M	68	Parietal	GBM, NOS	4	0		T2WI	0	1	
00018	F	74	Occipital	GBM, NOS	4	1	Immunostainig	FLAIR	1	1	
00019	M	75	Temporal	GBM, NOS	2	1	Immunostainig	T2WI	1	1	
00020	F	79	Frontal	GBM, IDH-wt	4	1	Sanger sequencing	T2WI	1	1	
00021	M	77	Temporal	GBM, NOS	1	1	Immunostainig	T2WI	0	1	
00022	M	58	Frontal	GBM, NOS	6	1	Immunostainig	FLAIR	1	1	
00023	F	68	Parietal	GBM, IDH-wt	2	1	Sanger sequencing	FLAIR	1	1	
00024	F	47	Frontal	GBM, IDH-wt	5	1	Sanger sequencing	FLAIR	1	1	
00025	M	63	Thalamus	GBM, NOS	1	0		T2WI	0	1	

Abbreviations: F, Female; M, Male; GBM, glioblastoma; wt, wild-type; NOS, Not otherwise specified
