# Supplementary material for: Radiomics Analysis of Non-Enhancing Lesions After Bevacizumab Administration in Recurrent Glioblastoma
Source: Bioengineering (Basel). 2025 Dec 26;13(1):28. doi: 10.3390/bioengineering13010028 (PMC12837343; doi:10.3390/bioengineering13010028)
Supplement: Supplementary file 1 [file bioengineering-13-00028-s001.zip › bioengineering-4005487-supplementary/bioengineering-4005487-supplementary/Supplementary Table S2.pdf]

Supplementary Table S2. Detailed demographic data for the Met-PET cohort.

Image ID	Sex	Age	Lesion	Diagnosis	IDH (wt:1, unknown:0)	IDH mutation diagnosis	Reference	FLAIR (Yes:1, No:0)	T2IW (Yes:1, No:0)	Met-PET (Yes:1, No:0)	Comment
00001	F	64	Parietal	GBM, IDH-wt	1	Sanger sequencing	FLAIR	1	1	1	
00002	M	70	Temporal	GBM, IDH-wt	1	Sanger sequencing	T2WI	0	1	1	
00003	M	63	Parietal	GBM, IDH-wt	1	Sanger sequencing	FLAIR	1	1	1	
00004	M	41	Frontal	GBM, IDH-wt	1	Sanger sequencing	FLAIR	1	1	1	
00005	M	66	Temporal	GBM, IDH-wt	1	Sanger sequencing	FLAIR	1	1	1	
00006	M	34	Temporal, Parietal	GBM, IDH-wt	1	Sanger sequencing	FLAIR	1	1	1	
00007	F	51	Temporal	GBM, IDH-wt	1	Sanger sequencing	FLAIR	1	1	1	
00008	F	68	Parietal	GBM, IDH-wt	1	Sanger sequencing	FLAIR	1	1	1	
00009	M	72	Temporal	GBM, IDH-wt	1	Sanger sequencing	FLAIR	1	1	1	
00010	M	75	Temporal	GBM, IDH-wt	1	Sanger sequencing	FLAIR	1	1	1	
00011	M	43	Temporal	GBM, IDH-wt	1	Sanger sequencing	FLAIR	1	1	1	
00012	M	67	Temporal	GBM, IDH-wt	1	Sanger sequencing	FLAIR	1	1	1	
00013	M	64	Frontal	GBM, IDH-wt	1	Sanger sequencing	FLAIR	1	1	1	Excluded
00014	M	71	Temporal	GBM, IDH-wt	1	Sanger sequencing	FLAIR	1	1	1	
00015	M	37	Frontal	GBM, IDH-wt	1	Sanger sequencing	FLAIR	1	1	1	
00016	F	52	Temporal	GBM, IDH-wt	1	Sanger sequencing	FLAIR	1	1	1	
00017	F	62	Temporal	GBM, IDH-wt	1	Sanger sequencing	FLAIR	1	1	1	
00018	F	83	Parietal, Occipital	GBM, IDH-wt	1	Sanger sequencing	FLAIR	1	1	1	
00019	F	34	Temporal	GBM, IDH-wt	1	Sanger sequencing	FLAIR	1	1	1	
00020	M	64	Frontal	GBM, IDH-wt	1	Sanger sequencing	FLAIR	1	1	1	
00021	M	52	Frontal	GBM, IDH-wt	1	Sanger sequencing	FLAIR	1	1	1	
00022	F	34	Frontal	GBM, IDH-wt	1	Sanger sequencing	FLAIR	1	1	1	
00023	F	73	Frontal	GBM, IDH-wt	1	Sanger sequencing	FLAIR	1	1	1	
00024	F	69	Frontal	GBM, IDH-wt	1	Sanger sequencing	FLAIR	1	1	1	
00025	F	54	Frontal	GBM, IDH-wt	1	Sanger sequencing	FLAIR	1	1	1	

Abbreviations: F, Female; M, Male; GBM, glioblastoma; wt, wild-type.
