# Supplementary material for: Radiomics Analysis of Non-Enhancing Lesions After Bevacizumab Administration in Recurrent Glioblastoma
Source: Bioengineering (Basel). 2025 Dec 26;13(1):28. doi: 10.3390/bioengineering13010028 (PMC12837343; doi:10.3390/bioengineering13010028)
Supplement: Supplementary file 1 [file bioengineering-13-00028-s001.zip › bioengineering-4005487-supplementary/bioengineering-4005487-supplementary/Supplementary Table S3.pdf]

Supplementary Table S3. The mean AUC, individual AUC, BH-adjusted p-values, p-values, and median values of the significant radiomic features across both cohorts.

Type	Mean AUC	BEV cohort					Met-PET cohort				
		ROC analysis	BH-adjusted P value	MWU test	Median		ROC analysis	BH-adjusted P value	MWU test	Median	
		AUC		P value	T2FLH	nCET	AUC		P value	T2FLH	nCET
**FLAIR_whole_Min**	0.875	0.848	<0.001	<0.001	36	61	0.903	<0.001	<0.001	55	13
**FLAIR_whole_GLRLMLrge**	0.834	0.892	<0.001	<0.001	169.4	187.4	0.777	0.007	<0.001	191.3	167.6
**T2WI_whole_GLCMcorrelation_1**	0.833	0.928	<0.001	<0.001	0.942	0.843	0.737	0.019	0.005	0.931	0.886
**FLAIR_whole_GLCMcorrelation_1**	0.831	0.957	<0.001	<0.001	0.945	0.848	0.706	0.041	0.018	0.931	0.897
**FLAIR_whole_GLCMcorrelation_2**	0.826	0.951	<0.001	<0.001	0.883	0.714	0.702	0.044	0.020	0.860	0.798
**T2WI_whole_GLCMcorrelation_2**	0.826	0.926	<0.001	<0.001	0.881	0.707	0.726	0.024	0.007	0.862	0.787
**T2WI_whole_GLCMcorrelation_3**	0.823	0.924	<0.001	<0.001	0.819	0.596	0.723	0.026	0.008	0.795	0.694
**T2WI_whole_GLCMcorrelation_1_SD**	0.817	0.917	<0.001	<0.001	0.014	0.033	0.716	0.033	0.011	0.015	0.026
**T2WI_whole_GLCMcorrelation_2_SD**	0.806	0.899	<0.001	<0.001	0.028	0.051	0.714	0.034	0.011	0.030	0.044
**T2WI_whole_GLRLMLrge**	0.763	0.781	0.003	0.001	136.5	153.2	0.745	0.015	0.003	157.8	133.3

Abbreviations: GLRLM, Gray Level Run Length Matrix; GLRLMLrge, Gray Level Run Length Matrix Low gray level run emphasis; GLCM, Gray Level Co-occurrence Matrix; SD, Standard deviation; ROC, receiver-operating characteristic; AUC, area under the ROC curve; MWU test, Mann-Whitney U test; BH, Benjamini–Hochberg.
